# Naturally-Acquired Dengue Virus Infections Do Not Reduce Short-Term Survival of Infected *Aedes aegypti* from Ho Chi Minh City, Vietnam

**DOI:** 10.4269/ajtmh.14-0499

**Published:** 2015-03-04

**Authors:** Lauren B. Carrington, Hoa L. Nguyen, Nguyet Minh Nguyen, T. H. Kien Duong, Trung Vu Tuan, Nguyen Thi Giang, Nhu Vu Tuyet, Dui Le Thi, Long Vo Thi, Chau N. Tran, Cameron P. Simmons

**Affiliations:** Oxford University Clinical Research Unit, Hospital for Tropical Diseases, Ho Chi Minh City, Vietnam; Department of Microbiology and Immunology, The University of Melbourne, Melbourne, Victoria, Australia; Centre for Tropical Medicine, Nuffield Department of Clinical Medicine, University of Oxford, Oxford, United Kingdom

## Abstract

Transmission of dengue virus (DENV) from mosquito to human is dependent upon the survival of the mosquito beyond the virus extrinsic incubation period. Previous studies report conflicting results of the effects of DENV on *Aedes aegypti* survival. Here, we describe the effect of DENV on the short-term survival (up to 12 d) of 4,321 *Ae. aegypti* mosquitoes blood-fed on 150 NS1-positive dengue patients hospitalized in the Hospital for Tropical Diseases, Ho Chi Minh City, Vietnam. Mosquito survival was not different between cohorts that fed upon blood from which 0% of mosquitoes became DENV infected (*N* = 88 feeds), or 100% became infected (*N* = 116 feeds). Subgroup analysis also did not reveal serotype-dependent differences in survival, nor a relationship between survival and human plasma viremia levels. These results suggest that DENV infection adds minimal cost to *Ae. aegypti*, an important finding when parameterizing the vector competence of this mosquito.

## Background

Dengue viruses (DENV) infected an estimated 390 million people in 2010,^1^ and dengue is widely considered the most common arboviral disease of humans. The transmission of DENV from mosquitoes to humans is, among other things, dependent upon the susceptibility of the mosquito to the infection, the time it takes for the virus to disseminate to the salivary glands for transmission, and mosquito survival. Vectorial capacity can be expressed by the equation:

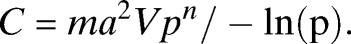


Here, *m* represents the relative abundance of vectors to humans (mosquito density), *a* is the daily biting rate of female mosquitoes, *V* is the innate susceptibility of the mosquito to the infection, *p* represents the daily probability of mosquito survival, and *n* is the extrinsic incubation period of the pathogen, measured in days. This value of *p*, mosquito probability of survival, is a critically important component of vectorial capacity. Low mosquito survival subsequent to DENV infection will reduce the likelihood of onward transmission of the infecting virus to a new host. Given the relatively long extrinsic incubation period of DENV compared with that of some other arboviruses (compare chikungunya virus ∼2–3 d post-exposure^2^,^3^; and yellow fever virus ∼4–6 d post-exposure),^4^ this emphasizes the importance of middle and old age classes of mosquitoes. Hence, understanding how DENV infection impacts vector survival is vital for improving the predictions of DENV transmission dynamics.

There has been overall inconsistency among the reports of the effects of arboviruses on mosquito vectors. An overall negative effect of arboviral infections on mosquito survival was shown in a meta-analysis across 16 studies involving various vector virus systems^5^; the direction of these effects, however, were dependent upon the specific system. Within the DENV-*Aedes aegypti* literature, some studies report negligible effects of DENV infection on mosquito survival, whereas others show reductions in mosquito lifespan. Sample sizes and experimental designs (using animal blood spiked with cultured virus for blood feeds, or the length of colonization of mosquitoes, for example) also may have influenced the magnitude of the observed effects.

More recent studies suggest that viral titer in the blood meal might play a role in *Ae. aegypti* survival, and may explain some of the discordance between studies.^6^ Higher viral titers in the blood meal led to increased mortality of *Ae. aegypti* after DENV-2 infection in recently collected field mosquitoes. The same authors also found that *Ae. aegypti* never exposed to DENV-2 have lower mortality rates than those that have been exposed to the virus, irrespective of their eventual infection status. However, of the experimental subset that was exposed to DENV-2, those that developed the infection had a higher survival than those that failed to do so. This was however, after an extended incubation period (> 35 d),^6^ an age much greater than most mosquitoes are expected to live in the field.^7^ Carrington and others^8^,^9^ obtained comparable results; mosquitoes with a detectable DENV-1 infection had higher survival rate than those that failed to develop an infection despite parallel exposure to the same virus. This effect was noted under some combinations of mean temperature and diurnal temperature ranges but not others, showing the major effect that temperature has on mosquito survival and susceptibility to DENV infection. To the contrary, Moutailler and others^10^ observed reduced survival in *Ae. aegypti* infected with DENV-2 after 14 d. It should be recognized that each of these studies typically examined *Ae. aegypti* survival after oral exposure to laboratory cultured DENV spiked into animal blood. How well this serves as a model of *Ae. aegypti* survival after they acquire DENV infection by feeding on a viremic human host is unclear.

Until now, no studies have explored the effects of naturally acquired DENV infection, across multiple serotypes, on mosquito survival. To this end, we studied the 12 d survival of cohorts of *Ae. aegypti* mosquitoes that fed directly on acute dengue patients and compared survival of blood-fed mosquito cohorts where 100% of mosquitoes became DENV-infected versus those where 100% remained uninfected. These survival results can inform modeling of DENV transmission dynamics, including entomological interventions such as *Wolbachia*, for dengue control.

## Materials and Methods

### Patient cohort.

Patients with ≤ 72 hr of fever who were NS-1 rapid test positive and presented at the Hospital for Tropical Diseases (HTD) in Ho Chi Minh City (HCMC), Vietnam, were enrolled into a descriptive study investigating the transmission of DENV from humans to mosquitoes as described previously by Nguyen and others.^11^ All patients provided written informed consent to participate in the study. The study protocols relevant to this work were reviewed and approved by the Scientific and Ethical committee of the HTD (CS/NĐ/09/24, CS/NĐ/12/15, and CS/NĐ/11/08) and the Oxford Tropical Research Ethical Committee (OxTREC 20–09, OxTREC 29–12, and OxTREC 40–11).

### *Aedes aegypti* for human to mosquito transmission studies.

As previously described, F_3_ *Ae. aegypti* collected from District 8, Ho Chi Minh City were fed on NS-1 rapid test positive dengue patients. Before patient feeding, the F_2_ generation of mosquitoes was tested by reverse transcription-polymerase chain reaction (RT-PCR) and confirmed as negative for DENV, chikungunya virus, and Japanese encephalitis virus infections. Colony maintenance was performed in an environmental chamber held at 27°C, 70% relative humidity, with 12 hr:12 hr light:dark. Females were provided blood meals from afebrile healthy volunteers for egg production. Further details can be found in Nguyen and others.^11^

### Experimental exposure of patients to *Aedes aegypti* mosquitoes.

As described by Nguyen and others,^11^ patients who had given informed consent were exposed on two occasions to *Ae. aegypti* mosquitoes during the first 4 days of enrolment. The randomly allocated schedule for exposure was blinded to the participant and investigators until the time point that the study number was assigned to the patient. At each allotted time point, the patient's forearm was exposed to between 25 and 40 pre-mated *Ae. aegypti* females, 3 to 7 days old, contained in a mesh-covered 350 mL plastic cup held against the patient's forearm for 5 min. After feeding, mosquitoes were cold anesthetized. Engorged mosquitoes were transferred to small plastic cups (maximum of 15 females per cup) and maintained in an environmental chamber, set at 27°C and 70% relative humidity, for the duration of the experiment.

### Mosquito survival experiments.

We analyzed the mosquito and patient data described by Nguyen and others^11^; detailed descriptions of patient characteristics can be found within this publication and the associated supplemental information, along with details of immature mosquito nutrition and rearing practices. Briefly, mosquitoes that fed upon NS-1 positive patients were collected and incubated for 12 d before being harvested and tested for DENV infection of abdomen tissues. Mosquito survival was monitored daily. The infection status of mosquito cohorts was established in the homogenized tissues of individual mosquito abdomens by RT-PCR as per protocols described in Nguyen and others.^11^ and Duong and others.^12^ All laboratory assays of mosquitoes were performed by technicians blinded to the clinical and virological details of the patients.

### Dengue diagnostics.

The DENV plasma viremia levels were measured by a validated, quantitative RT-PCR assay that has been described previously.^12^ Venous blood draws were taken from patients within 30 mins of each feed. The mosquito blood meal was determined as either negative or positive for DENV using RT-PCR; positive samples had the viremia recorded (log_10_ viral RNA copies/mL). The RT-PCR assay was calibrated against the infectious virus (grown in mammalian BHK-21 cells), and the ratio between genome copies per mL and plaque-forming units per mL was 214:1 for DENV-1, 73:1 for DENV-2, 436:1 for DENV-3, and 101:1 for DENV-4.

### Inclusion/exclusion criteria for mosquitoes.

Survival analyses included data from female cohorts exposed to DENV RT-PCR positive blood and were either 100% or 0% DENV-infected 12 d after blood feeding, under the assumption that those mosquitoes that died before the sampling day (Day 12) from these cohorts were of the same infection status as other mosquitoes. We also included data from mosquitoes exposed to DENV RT-PCR-negative patients (although patients were NS-1 positive at the time of enrollment in the study).

We excluded from the analysis cohorts of females where the DENV infection rate was between 1% and 99% because in these cohorts we could not be certain of the DENV infection status of mosquitoes that died before Day 12. We also excluded those cohorts in which some mosquitoes were harvested before the 12 d time point, and also those cohorts that had fewer than five engorged females.

### Statistical analysis.

Characteristics of the mosquito population were described using proportions for categorical variables (e.g., infection status, serotype) and medians (interquartile ranges [IQR]) for continuous variables (e.g., number of mosquitoes per feeding, plasma viremia level). Kaplan Meier (KM) survival estimates were created according to RT-PCR results (negative versus positive), and the infection status of mosquitoes (0% versus 100%) for all mosquitoes and stratified by infection serotype. In examining the association between infection status and mosquitoes survival, unadjusted and adjusted Cox proportional hazard (PH) regression models that take into account clustered data within patients were performed to obtain hazard ratios (HRs) and 95% confidence intervals (CIs). Potential confounding factors for this association include DENV serotype, and plasma viremia of the infectious blood meal. Proportional hazard assumptions were checked graphically by plotting a Cox model versus Kaplan-Meier curves, and the assumptions of these models were satisfied (data not shown). All analyses were performed using STATA v11.0 (StataCorp., College Station, TX).

## Results

Mosquito survival data from 204 eligible cohorts of *Ae. aegypti* (total 4,321 mosquitoes) who fed upon 150 Vietnamese dengue patients were analyzed. Table 1 describes the characteristics of the blood-fed cohorts of the mosquitoes in this study. These mosquitoes represented 48.6% of the mosquitoes that were blood-fed in the study described by Nguyen and others.^11^ A flowchart summarizing the exclusions (patients and associated mosquitoes) can be found in Supplemental Figure 1. The median plasma viremia that mosquitoes were exposed to for infectious blood meals was 8.5 log_10_ DENV RNA copies/mL of blood (*N* = 116, IQR: 7.8–9.0 log_10_ DENV RNA copies/mL; Table 1). The greater proportion of mosquito exposures were to DENV-1 and DENV-2, which were the predominant serotypes in circulation in Ho Chi Minh City at the time of the study.

### Effect of DENV infection on 12 d survival.

**Figure 1.**
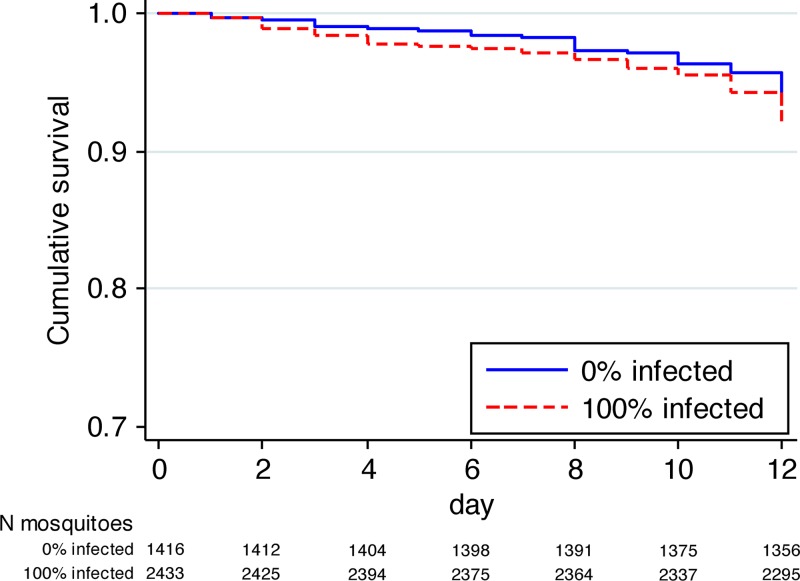
Cumulative survival over 12 days of dengue virus (DENV)-infected and uninfected females that had blood-fed on dengue patients. The number of mosquitoes still alive (remain “at risk” of dying) at each time point for the 0% infected and 100% infected groups are indicated below the graph.

We examined the 12 d survival in patient blood-fed mosquito cohorts in which 100% of members became DENV-infected (*N* = 116 cohorts), versus those where 100% of mosquitoes remained uninfected (*N* = 88). We found no significant difference in the survival of *Ae. aegypti* between these two groups, in both unadjusted (HR: 1.00; 95% CI = 0.44–2.27, *P* = 0.99) and adjusted analyses (HR: 1.18; 95% CI = 0.54–2.59, *P* = 0.68; Figure 1). These data suggest DENV infection does not change the probability of *Ae. aegypti* survival over a 12 d period in laboratory conditions.

### Effect of individual DENV serotypes and patient viremia on 12 d survival in exposed females.

**Figure 2.**
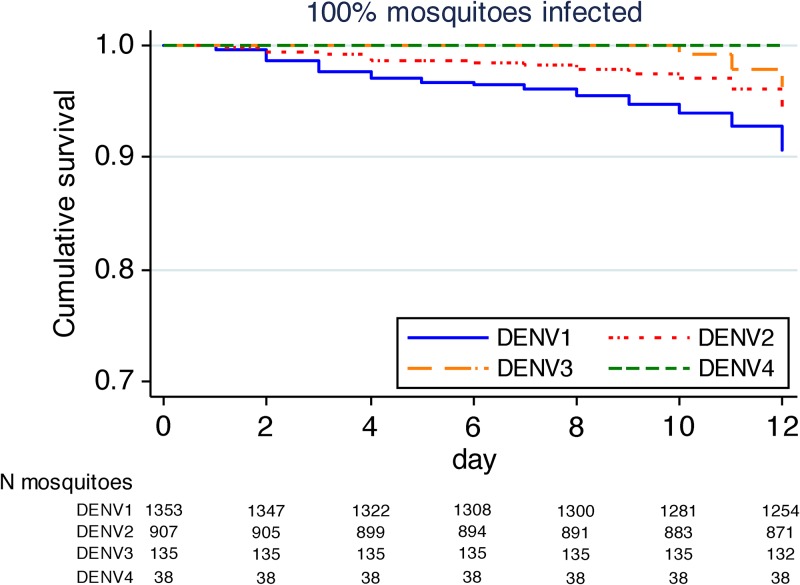
Cumulative survival over 12 days of dengue virus (DENV)-infected females that had blood-fed on dengue patients, stratified for each DENV serotype. The number of mosquitoes still alive at each time point and serotype is indicated below the graph.

We did not observe any inter-serotype differences in survival after exposure to each of the four DENV serotypes (Figure 2

; Table 2). Stratification by serotype also failed to show any differences in survival between infected and uninfected mosquitoes within the DENV-1 and DENV-2 serotypes (data not shown). There were insufficient numbers of mosquitoes exposed to DENV-3 and DENV-4 to adequately measure the serotype-specific effect of the infection on adult survival for these groups.

Results from multivariable adjusted PH Cox model showed that there was no significant effect of higher plasma viremia on mosquito survival (adjusted HR for +1 log10 RNA copies/mL: 0.98, 95% CI = 0.82–1.18 *P* = 0.85; Table 2). These results suggest that although more mosquitoes become infected with higher plasma viral titers, the survival of those infected *Ae. aegypti* does not differ from those that do not become infected.

## Discussion

Results from this study show no detectable effect of naturally-acquired DENV on female *Ae. aegypti* survival up to 12 d post-blood feeding. After stratification by serotype, we similarly did not detect a serotype-related effect on adult survival. This is in contrast with several recent studies that have shown variable effects of DENV on *Ae. aegypti* survival.^6^,^8^–^10^ The differences may be partially explained because each of these studies used artificial feeding systems, and virus grown in cell culture with variable blood meal titers between studies, and it is uncertain if the results mimic real-world pathogenesis of DENV infections, and that of other arboviruses in *Aedes* mosquitoes in the field. Although in this current study, mosquitoes were fed directly on viremic DENV patients, with each of the four DENV serotypes represented across a range of viremia values. In this “natural history” context, using a large sample size of mosquitoes that almost doubles that of the previous four studies combined, we failed to identify significant changes in mosquito survival in response to DENV infection.

### Survival effects on vectorial capacity.

Vector survival has a large influence on virus transmission potential. One plausible outcome of mosquito exposure to DENV is that the mosquito acquires a transient infection that it actively suppresses and eliminates. It has been hypothesized that the cost of inhibiting an infection might be greater than simply letting the virus establish and infect the organism.^6^,^8^,^13^ However, our results suggest that this is not the case. Accordingly, DENV-exposed mosquitoes in nature have the same life expectancy as those that have never imbibed any virus. For calculations of vectorial capacity, this implies that we can justifiably maintain a simplified estimate of survival for both infected and uninfected mosquitoes in calculations of vectorial capacity.

### Study limitations.

A limitation of this study is that survival was only monitored for a period of 12 d, rather than the entire lives of the mosquitoes. However, both laboratory^14^ and field-based estimates of *Aedes* survival^15^–^17^ suggest that *Ae. aegypti* senesce with age. Indeed, age-grading techniques applied to mosquitoes from the Southwestern United States estimate that only 9% of field females survive to 15 d of age.^7^ What remains unknown is whether natural DENV infection influences survival later in life. An assumption of this work is that the fate of those mosquitoes that were not tested for infection status (those that died before harvesting at Day 12 post-exposure) were of the same infection status (at Day 12) as other mosquitoes within their cohort. We specifically excluded those cohorts that had infection rates other than 0% or 100%, as we could not be confident of the infection status of mosquitoes that had died. Nevertheless, all estimates of *Ae. aegypti* survival reported here should be considered as conservative, as the standard laboratory conditions and nutrition provided to mosquitoes would be more agreeable than found in the wild.

## Conclusion

This is the first study to assess the survival of field-derived *Ae. aegypti* after acquisition of DENV infection. These unique results showed an overall negligible effect of DENV on the survival of *Ae. aegypti*. Thus, predictions of DENV transmission may consider infected and uninfected *Ae. aegypti* to have the same survival when naturally infected with the virus, at least until the time that they become infectious. Further studies are needed to elucidate whether virus-inhibiting *Wolbachia* alters this relationship, and what happens in field-like conditions as these mosquitoes begin to senesce.

## Supplementary Material

Supplemental Figure.

## Figures and Tables

**Table 1:** Plasma viremia according to infection status and serotype

Serotype	Number of patients	Number of feeding events*	Total no. of mosquitoes	Median of mosquitoes (IQR) per feeding	Number (%) of feedings with 0% infected	Number (%) of feedings with 100% infected	Plasma viremia (median, IQR) log 10-copies/mL		
							All (*n* = 204 feeds)	0% Infected (*N* = 88 feeds)	100% Infected (*N* = 116 feeds)
None	18	20	472	25 (22–30)	20 (100)	0 (0)	< LOD†	N/A	N/A
DENV-1	58	82	1,727	21 (17–27)	20 (24.3)	62 (75.6)	8.3 (6.9–9.1)	5.2 (4.1–5.8)	8.7 (7.9–9.2)
DENV-2	54	78	1,645	21 (17–25)	33 (42.3)	45 (57.6)	7.0 (5.0–8.2)	4.8 (4.4–5.3)	8.2 (7.2–8.6)
DENV-3	10	14	254	19 (12–22)	7 (50.0)	7 (50.0)	7.8 (6.5–8.9)	5.0 (4.9–7.7)	8.9 (8.7–9.4)
DENV-4	10	10	223	24.5 (16–27)	8 (80.0)	2 (20.0)	5.9 (5.7–7.6)	5.9 (5.7–7.3)	7.6 (7.6–8.3)
All	150	204	4,321	24 (19–28)	88 (43.14)	116 (56.86)	7.7 (5.7–8.7)	5.2 (4.4–5.9)	8.5 (7.8–9.0)

* Data from feedings with < 5 mosquitoes, and those from infection rates > 0% and < 100% were excluded.

†< LOD = below limit of detection of reverse transcription-polymerase chain reaction (RT-PCR) assay.

DENV = dengue virus; IQR = interquartile range.

**Table 2:** Associations between mosquito infection status and mosquito survival after exposure to all four DENV serotypes

Variables	Unadjusted		Adjusted	
	Hazard ratio (95% CI)	*P* value	Hazard ratio (95% CI)*	*P* value
Infection status				
0% infection	1.00 (reference)		1.00 (reference)	
100% infection	1.31 (0.80–2.16)	0.28	1.17 (0.57–2.40)	0.66
Serotype				
DENV-1	1.00 (reference)		1.00 (reference)	
DENV-2	0.62 (0.38–1.02)	0.06	0.63 (0.40–1.00)	0.051
DENV-3	0.72 (0.39–1.33)	0.30	0.75 (0.40–1.40)	0.37
DENV-4	0.38 (0.11–1.35)	0.14	0.41 (0.12–1.49)	0.18
Plasma viremia (+1 log_10_ copies/mL)	1.06 (0.93–1.21)	0.40	0.98 (0.82–1.18)	0.85

* Hazard ratios from Cox proportional hazard models adjusting for clustering data within patients. All variables in the table were included in multivariable models.

DENV = dengue virus; IQR = interquartile range.

## References

[1] Bhatt S, Gething PW, Brady OJ, Messina JP, Farlow AW, Moyes CL, Drake JM, Brownstein JS, Hoen AG, Sankoh O, Myers MF, George DB, Jaenisch T, Wint GR, Simmons CP, Scott TW, Farrar JJ, Hay SI (2013). The global distribution and burden of dengue. Nature.

[2] Dupont-Rouzeyrol M, Caro V, Guillaumot L, Vazeille M, D'Ortenzio E, Thiberge JM, Baroux N, Gourinat AC, Grandadam M, Failloux AB (2012). Chikungunya virus and the mosquito vector *Aedes aegypti* in New Caledonia (South Pacific Region). Vector Borne Zoonotic Dis.

[3] Dubrulle M, Mousson L, Moutailler S, Vazeille M, Failloux AB (2009). Chikungunya virus and *Aedes* mosquitoes: saliva is infectious as soon as two days after oral infection. PLoS ONE.

[4] Davis NC (1932). The effect of various temperatures in modifying the extrinsic incubation period of the yellow fever virus in *Aedes aegypti*. Am J Epidemiol.

[5] Lambrechts L, Scott TW (2009). Mode of transmission and the evolution of arbovirus virulence in mosquito vectors. Proc Biol Sci.

[6] Maciel-de-Freitas R, Koella JC, Lourenço-de-Oliveira R (2011). Lower survival rate, longevity and fecundity of *Aedes aegypti* (Diptera: Culicidae) females orally challenged with dengue virus serotype 2. Trans R Soc Trop Med Hyg.

[7] Joy TK, Gutierrez EH, Ernst K, Walker KR, Carriere Y, Torabi M, Riehle MA (2012). Aging field collected *Aedes aegypti* to determine their capacity for dengue transmission in the Southwestern United States. PLoS ONE.

[8] Carrington LB, Seifert SN, Armijos MV, Lambrechts L, Scott TW (2013). Reduction of *Aedes aegypti* vector competence for dengue virus under large temperature fluctuations. Am J Trop Med Hyg.

[9] Carrington LB, Armijos MV, Lambrechts L, Scott TW (2013). Fluctuations at low mean temperatures accelerate dengue virus transmission by *Aedes aegypti*. PLoS Negl Trop Dis.

[10] Moutailler S, Guichoux E, Vazeille M, Failloux AB (2010). Differential mortalities of dengue-infected *Aedes aegypti*: preliminary results. Ann Soc Entomol Fr.

[11] Nguyen NM, Kien DT, Trung VT, Quyen NT, Chau TN, Lon VT, Dui LT, Nguyen HL, Farrar J, Holmes EC, Rabaa MA, Bryant JE, Truong NT, Huong NT, Lan NT, Mai PP, Hung NT, Tai LT, Wills B, Chau NV, Wolbers M, Simmons CP (2013). Host and viral features of human dengue cases shape the population of infected and infectious *Aedes aegypti* mosquitoes. Proc Natl Acad Sci USA.

[12] Duong TH, Vu TT, Hanh TN, Tran Ngyuen BC, Huynh LA, Wills BA, Simmons CP (2011). Validation of an internally controlled one-step real-time multiplex RT-PCR assay for the detection and quantitation of dengue virus RNA in plasma. J Virol Methods.

[13] Schmid-Hempel P (2005). Evolutionary ecology of insect immune defenses. Annu Rev Entomol.

[14] Styer LM, Carey JR, Wang J-L, Scott TW (2007). Mosquitoes do senesce: departure from the paradigm of constant mortality. Am J Trop Med Hyg.

[15] Harrington LC, Vermeylen F, Jones JJ, Kitthawee S, Sithiprasasna R, Edman JD, Scott TW (2008). Age-dependent survival of the dengue vector *Aedes aegypti* (Diptera: Culicidae) demonstrated by simultaneous release-recapture of different age cohorts. J Med Entomol.

[16] Muir LE, Kay BH (1998). *Aedes aegypti* survival and dispersal estimated by mark-release-recapture in northern Australia. Am J Trop Med Hyg.

[17] Harrington LC, Buonaccorsi JP, Edman JD, Costero A, Kittayapong P, Clark GG, Scott TW (2001). Analysis of survival of young and old *Aedes aegypti* (Diptera: Culicidae) from Puerto Rico and Thailand. J Med Entomol.

