# Supplementary material for: Naturally-Acquired Dengue Virus Infections Do Not Reduce Short-Term Survival of Infected Aedes aegypti from Ho Chi Minh City, Vietnam
Source: Am J Trop Med Hyg. 2015 Mar 4;92(3):492–6. doi: 10.4269/ajtmh.14-0499 (PMC4350536; doi:10.4269/ajtmh.14-0499)
Supplement: Supplementary file 1 [file SD3.pdf]

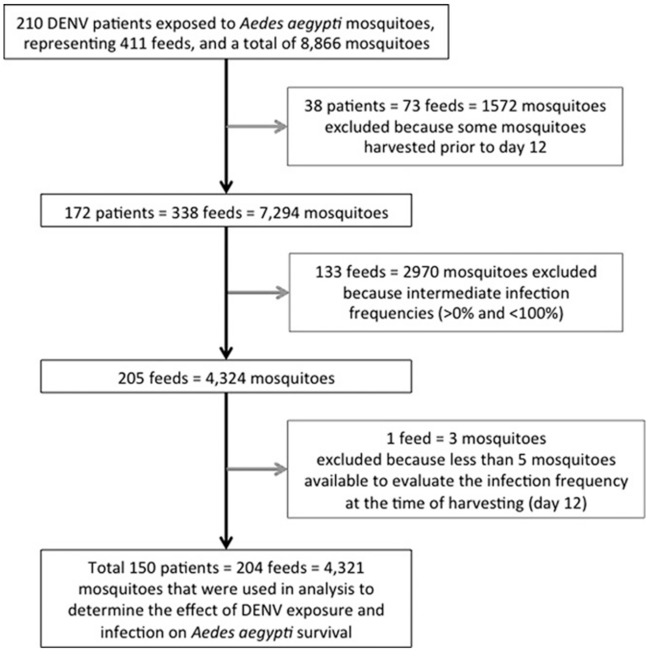

Supplemental Figure 1. Flowchart illustrating process and progression patient enrollment, and mosquito exposures and sample inclusion/exclusion for statistical analysis.
